# Quantification of the Photocatalytic Self-Cleaning Ability of Non-Transparent Materials

**DOI:** 10.3390/ma12030508

**Published:** 2019-02-08

**Authors:** Marco Minella, Claudio Minero

**Affiliations:** Department of Chemistry and NIS Center of Excellence, University of Torino, Via P. Giuria 5, 10125 Torino, Italy; marco.minella@unito.it

**Keywords:** self-cleaning, photocatalytic materials, solid state reactivity, methylene blue, rhodamine B, metanil yellow, titanium dioxide, iron oxide, smart materials

## Abstract

The photo-induced reactivity of compounds at the surface of photocatalytic materials is used to maintain the cleanliness of the surface of glass, concretes and paints. A standard method to quantify the photocatalytic self-cleaning (SC) properties of non-transparent materials was recently published. It is based on the covering of the sample surface with a defined amount of dye and on the evaluation of the reflectance spectra of the coloured surface under irradiation. The calibration of the spectral changes allowed the quantification of the surface residual dye and the evaluation of the self-cleaning kinetics. The method was tested on seven white and coloured photocatalytic materials using methylene blue (MB), rhodamine B (RhB) and metanil yellow (MY). The main by-products of the MB photocatalytic degradation at the solid/solid interface were identified, showing that MB degradation in solution follows a path quite different from that at the solid/solid interface. Also MY showed a different order of photoreactivity. Furthermore, experiments at the solid/solid interface are more trustworthy than tests in solution for evaluating the self-cleaning ability. The differences of the photocatalytic phenomena at the solid/solid interface in comparison with the most studied photoactivated processes at the solid/liquid interface are outlined. Furthermore, photocatalytic materials showed selectivity toward some specific dyes. This encourages the use of more than one dye for the evaluation of the self-cleaning ability of a photocatalytic material.

## 1. Introduction

The effects activated at the surface of a semiconductor following the absorption of photons with energy higher than its band gap (hν ≥ E_g_) have a wide range of potential environmental applications. The photocatalytic process under irradiated semiconductors has been widely investigated for several kinds of applications, like photovoltaics [1,2,3], hydrogen production by water photosplitting or photoreforming [4], degradation of recalcitrant organic and inorganic pollutants [5,6,7,8] and the synthesis of value added chemicals [9,10,11,12,13], but also for functional coatings with self-cleaning (SC) and self-sterilizing surfaces [14,15,16]. The insights into the photocatalytic process have undergone a successful transfer from the academic labs to the industrial fields. This process is mainly driven by the production of air purification systems and self-cleaning surfaces. The latter is perhaps the most important market for the photocatalytic materials, as photocatalytic glasses, concretes and pigments have major market value, which is growing constantly [17,18]. The self-cleaning properties of a material are related to the synergistic effect of the photocatalytic degradation and, ultimately, mineralization of organic compounds adsorbed at the surface, and the photoinduced superhydrophilicity of the TiO_2_ surface. A soft washing (e.g., rain) of the self-cleaning materials allows the dirt residues to be washed away resulting in a clean surface [19].

The internationally recognized standard methods to measure the photocatalytic activity of materials do not completely satisfy the needs of this growing market. The reported methods often do not fit with their specific purposes because the quantification of the measured feature is done either indirectly (e.g., measurement of the contact angle after UV irradiation to evaluate the self-cleaning ability of a glass) or under different conditions (e.g., measurement of the photocatalytic decolouring activity in solution to deduce the self-cleaning performance at the solid/solid interface). Worth noting among these methods are: (*i*) the measurement of the decolouring kinetics of dyes (methylene blue, acid orange 7, eosin Y, rhodamine B) in solution in contact with the irradiated photocatalytic material [20,21,22,23,24,25,26,27]; (*ii)* the measurement of the degradation rate of terephthalic acid in solution in contact with a photocatalytic surface by monitoring the fluorescence signal of its high fluorescent by-product hydroxyterephthalic acid [19,27]; (*iii*) the evaluation of the photocatalytic degradation of stearic acid spread on the catalytic surface through FT-IR spectroscopy or by measuring the variation of the water contact angle [22,28]; (*iv*) the evaluation of the colour change and disappearance of organic dyes (e.g., resazurin) in a solid polymer matrix deposited over the tested material [20,27,29,30,31]. The proposed methods have been mainly applied to evaluate the self-cleaning ability of photocatalytic glasses, the most promising commercial application of photocatalysis from an industrial point of view [17]. Tests to evaluate and quantify the SC properties of pigments, concretes or non-transparent photocatalytic products are limited both in the scientific literature and in standard methods. The only exception is the European Norm EN 16845-1:2017 [32], in which the degradation of dry dyes on photocatalytic surfaces is measured by reflectance spectroscopy. 

This norm, approved by the Comité Européen de Normalisation (CEN) committee TC386, details the procedure to quantify the self-cleaning activity of non-transparent and porous surfaces (concrete, photocatalytic fabrics, cement...). The norm is based on illumination of the tested surface dirtied with dry dye, obtained through spraying dye solutions (simulating soiling solutions) in controlled conditions. Then the sample is UV irradiated in air and the dye disappearance quantified by UV-Vis reflectance spectroscopy. The EN standard uses three different organic dyes—methylene blue (MB), rhodamine B (RhB) and metanil yellow (MY). The rationale for the choice of three different dyes for the self-cleaning test is based on the need to cover completely the visible range and then to have a sufficient optical contrast with possible coloured samples, or at least with one or two of them. On black/very dark surfaces, the method is not applicable because the reflectance is too low [32].

Here we report the experimental evidence, its discussion and relevance, which support and limit the application of the EN standard. We tested seven different photocatalytic materials (TiO_2_, barite and goethite) using the three dyes. Furthermore, we considered the differences between the photocatalytic reactivity at the solid/solid interface and solution. This topic is noteworthy for the measurement of a self-cleaning photocatalytic performance, even if it has had scarce attention up to now.

## 2. Materials and Methods

Tested samples were prepared on 10 cm × 10 cm pyrex glass substrates by the Doctor Blade method of water slurries of the photocatalytic powders reported in Table 1. Layers of infinite optical depth were obtained. The films were then dried in an oven at 100 °C for 10 min. The thickness of the films was in the micrometre range (roughly 1–5 µm) and depended on the nature of the powder.

The samples were prepared with a scope to produce photocatalytic samples of different natures to simulate possible real-life and commercial samples. For this scope, the materials were deliberatively chosen to be quite different. The experimental materials are intended to be illustrative of possible self-cleaning materials and of the reactivity of dyes at the solid/solid interface compared to that in solution. In this light, the specific properties of the materials, as well as their physico-chemical properties, are not important.

Methylene blue, rhodamine B and metanil yellow were purchased from Sigma-Aldrich and acetone from Carlo Erba Reagenti. All reagents were used as received. The absorption spectra of the studied organic dyes in acetone are reported in the Supplementary Material, Figure S1. They were acquired with a Varian Cary 100 Scan UV-Vis spectrophotometer, using quartz cuvettes with a path length of 1 cm. Table 2 reports the maximum absorption wavelengths and the molar extinction coefficient in acetone for the tested dyes.

A homemade spraying system was built according to the EN standard to perfectly control the spraying conditions. This system allowed maximising the homogeneity of the dye covering and modifying the amount of solution sprayed on the surface. The dye solutions (5 × 10^−4^ M in acetone for all the dyes) were spread using a spraying gun (Anest Iwata WA 101 E2P). The samples were placed orthogonally to the spraying flow direction. The spraying distance and orientation between the gun and the sample were chosen to obtain a homogeneous covering of the sample surface. The distance between the gun and the sample was fixed to 25 cm for all the tests. The solution to be sprayed and the atomization air pressure were at 3 ± 0.1 bar. Following EN standard the deposition rate (DR) was calculated. In the adopted conditions DR was 3 × 10^−6^, 3.8 × 10^−6^ and 3 × 10^−6^ g·s^−1^·cm^−2^ for MB, RhB and MY, respectively. The amount of solution spread was controlled by changing the spraying time with a digital timer (Omron, H5CX-A-N) that opened and closed the actuator line with a precision of ±0.01 s. 

**Figure 1:**
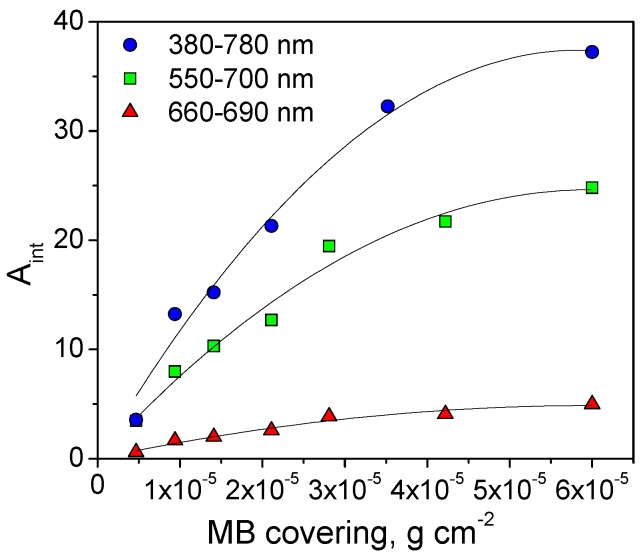
Calibration function of methylene blue (MB) on P25 TiO_2_: A_int_ calculated considering three different integration range (380–780 nm, 550–700 nm and 660–690 nm) as a function of the MB covering with the corresponding quadratic fit function.

**Figure 2:**
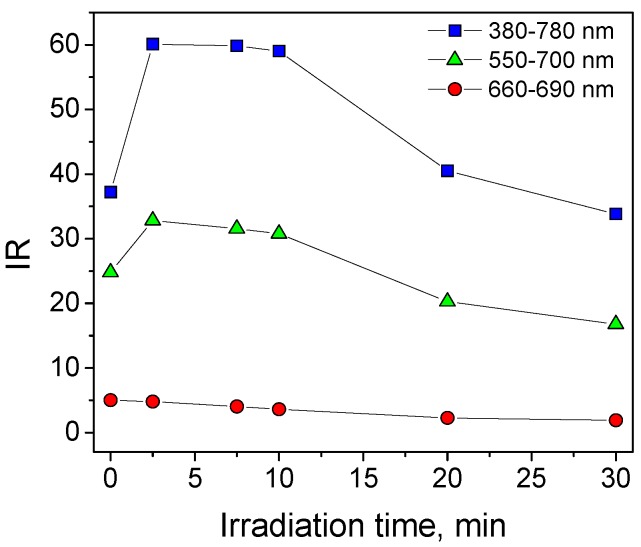
MB self-cleaning test on TiO_2_ P25: Integrated Reflectance (IR) calculated considering three different integration ranges as a function of the irradiation time. Note that only for IR in the 660–690 nm range can the IR profile be described as an exponential decay. SC = 4 × 10^−5^ g·cm^−2^.

**Figure 3:**
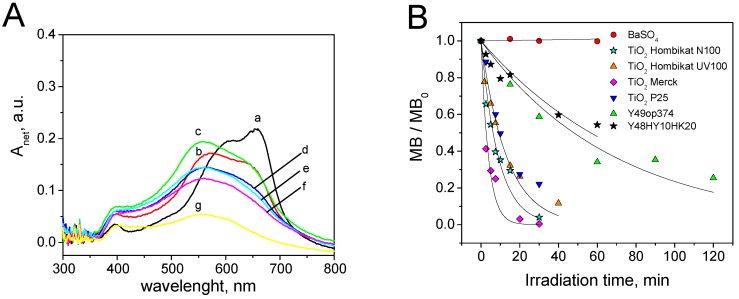
Self-cleaning test with methylene blue (MB): (**A**) Evolution of the surface spectra at different irradiation times (a = 0 min, b = 2.5 min, c = 5 min, d = 7.5 min, e = 10 min, f = 15 min, g = 30 min) during the MB self-cleaning test on TiO_2_ Hombikat N100; (**B**) Time evolution of relative surface concentration observed for MB on the seven tested photocatalytic materials. SC = 4 × 10^−5^ g·cm^−2^.

From the volume of dye solution spread on the sample in different steps, the amount of dye deposited (DP) on the surface was calculated. From the reflectance measurements at each step, an integral value of absorbance (A_int_) was determined and a calibration function was calculated (A_int_ as a function of dye covering DC, calculated from DP). The integration interval over a specific wavelength range typical of each dye was chosen to avoid spectral interference from by-products and to maximize the S/N ratio (see Table 2). As an example, Figure 1 shows the calibration function of MB over P25 TiO_2_ computed with a different interval of integration. This calibration was used to assess the residual amount of dye on the surface after its disappearance under irradiation. The calibration function is not linear with the deposited dye amount, as absorbance does not vary linearly with the dye surface concentration [33]. When the integration interval is large, a large value is obtained, although this can be biased by the formation of by-products that contribute to the reflectance (see Figure 2 and Figure 3A for MB). The proper integration interval must then be considered.

Figure 2 shows the evolution of A_int_ calculated considering three different wavelength integration ranges as a function of the irradiation time on TiO_2_ P25. The evolution of *A_int_^380–800 nm^* (e.g., interval of integration 380–800 nm) during the MB self-cleaning test showed a maximum in the first minutes of irradiation as a consequence of the formation of coloured intermediates, and then a monotonic decrease caused by the degradation of both MB and its intermediates. Limiting the interval of integration to 660–690 nm the A_int_ profile showed a monotonic decrease. Then the interval of integration was chosen to avoid the interference from coloured by-products and to maximise the S/N ratio at the same time. Then, measurement using colour measurement instruments, also with diffused illumination integrating spheres, and giving L*, a* and b* colorimetric coordinates, can lead to misleading conclusions with regard to the dye disappearance rate.

The absorption spectra of the dyes spread over the surface of the seven tested materials were only partially affected by the peculiar properties of the materials, as is shown by Figure S2, where the absorbance spectra of the dyes over the different tested materials before irradiation are compared. The spectra are quite different from those in acetone solution, as can be seen from the comparison of Figures S1 and S2. The MB spectrum shows a relevant blue shift, suggesting a strong interaction with all tested materials. Conversely, RhB and MY spectra are only little changed in the region of maximum absorption, and their spectral shape are slightly affected by the nature of the adsorbing material. As the used materials have similar surface acid-base properties (the pzc of barite, TiO_2_ and Goethite are 5.2 [34], 6.2 [35] and ≈7.5 [36], respectively), a marginal role of the different surface acidity on the protonation state of the dye is expected, and consequently a minor modification of the absorption spectra. The different textures of the powders affected the porosity and reflectance of the films, because of a possible different infiltration of the dye. This only affected the intensity of absorption of the dyes on the surface. Because the dye can penetrate inside the porous catalyst, the adopted spraying procedure would minimize this effect compared with an impregnation from solution. Moreover, the experimental procedure adopted (see below) is based on the evaluation of the relative amount of the dye remaining after a given irradiation time compared with the initial amount. In this light, the difference in the maximum absorbance or slight differences in the spectral shape do not affect the accuracy of the measurements.

The irradiation was carried out through a set of three TLK 40W/05 fluorescence lamps (Philips). The irradiance on the sample surface was 20.5 W·m^−2^ (between 290 and 400 nm). Figure S3 shows the lamp emission spectrum (emission band centred at 360 nm with a width at half maximum of 30 nm). During the irradiation the sample temperature was 25 ± 3 °C. The reflectance spectra of the sample surfaces were measured by means of an Ocean Optics USB2000 spectrophotometer with an external UV-Vis light source (Micropack DH-2000). The acquisition and elaboration of the spectra were made with the Ocean Optics Spectra Suite software. The spectra were recorded by using a diffuse reflectance standard of polytetrafluoroethylene (Ocean Optics WS-1-SS), the acquisition time for each scan was 5 s, each spectrum was the average of 5 scans, and the spectra were always the average of at least 4 spectra recorded in four different positions near the centre of the samples.

The identification of the main MB by-products during the self-cleaning experiments (at the solid/solid interface) was carried out by extracting, with water, the organic residues present at the sample surface after different irradiation times and analysing the sample by a HPLC equipped with a diode array detector and a mass spectrometer with an electrospray interface (HPLC-DAD-ESI-MS). The HPLC analysis was performed by means of a Thermo Finnigan instrument with a Lichrospher R100-CH 18/2 column (250 mm, 10 mm diameter) and a mobile phase composed of methanol–ammonium acetate 5 mM (isocratic mode, 30/70 v/v, pH 4.5, flow rate 1 mL·min^−1^). The detection was carried out through a UV–Vis diode array detector (Thermo Finnigan Surveyor PDA) and a Thermo Finnigan Surveyor MSQ mass spectrometer equipped with an ESI interface. MS analysis was performed in positive mode; the mass range was 100–1400 *m*/*z*. High purity nitrogen was used as nebulizer gas (5 bar). The ESI needle potential was set at 3 kV. The heated capillary was set to 300 °C and the cone voltage to 30 V.

The experiments in solution with MY were carried out on 5 mL of aqueous suspension with 0.5 g·dm^−3^ of photocatalyst and MY (1 × 10^−4^ M) with the same irradiation set up adopted for the self-cleaning tests. After irradiation, the suspensions were filtered through a 0.45 μm cellulose acetate membrane filter (Millipore HA) and analysed with a UV-Vis spectrophotometer. The entire apparatus is described elsewhere [37].

## 3. Results and Discussion 

### 3.1. Methylene Blue Self-Cleaning Test

MB is a thiazine dye often used as standard to evaluate the photocatalytic activity. The photocatalytic transformation of MB over irradiated TiO_2_ can occur both via the oxidative path Equation (1), up to the complete mineralization of the substrate to CO_2_ and mineral ions [38], and via the reductive process Equation (2). The latter path gives the leuco form of the dye (LMB) that is rapidly oxidized by the dissolved oxygen (Equation (3)) [39,40].

MB + n h_vb_^+^ (^∙^OH) →→→ CO_2_ + NO_3_^−^ + SO_4_^−2^ + NH_4_^+^ (1)

MB + 2e_cb_^−^ + H^+^ → LMB (2)

2LMB + O_2_ → MB + H_2_O (3)

The oxidative path is prevalent in aerobic neutral/alkaline conditions, while the reductive path is the main process in anaerobic acid conditions [39,40]. The reductive path is possible on thermodynamic grounds because the redox potential for the MB/LMB couple is 0.53 V vs NHE at pH 0 [22], while the photogenerated CB electrons at the same pH are placed roughly at –0.1 V vs NHE [1,41,42]. During the self-cleaning test here reported, the presence of oxygen hindered the production of the leuco (reduced) MB form for two reasons: (*i*) atmospheric O_2_ scavenged almost entirely the conduction band electrons; (*ii*) the possible MB leuco form reacted immediately with O_2_ to give the original colored MB according to Equation (3). The role of the leuco forms in the studied self-cleaning processes was consequently negligible. 

Figure S4 shows the reflectance spectra, reported as net absorbance in the 300–800 nm range, recorded during the MB self-cleaning test on barite (BaSO_4_). Barite does not show any photocatalytic activity. No evolution of the reflectance surface spectra under irradiation was observed. Neither direct photolysis nor thermal degradation of MB on BaSO_4_ was observed. As a consequence, during the SC test with MB, any changes in the reflectance spectra could be entirely ascribed to the photocatalytic properties of the sample (including the possibility of self-sensitization, i.e., charge injection from the excited state of the dye to the semiconductor conduction band).

Figure 3A shows the reflectance spectra at different irradiation times measured during the methylene blue SC test on TiO_2_ Hombikat N100. In this case, an evolution of the spectra as a function of the irradiation time was observed until an almost complete disappearance of the colour after 30 min of irradiation. The evolution of the spectra showed in this case the formation of coloured by-products with a maximum centred at 555 nm (see above for its relevance). 

The remaining dye at the surface was computed with the corresponding calibration curve obtained on the selected substrate with the proper wavelength integration interval (see for example Figure 1 obtained for TiO_2_ P25). The ratio between MB at the irradiation time *i* (*MB_i_*), and the initial covering (*MB*_0_), showed an exponential decay (Equation (4)):

(4) $$\frac{MB_{i}}{MB_{0}}=\exp\left(-k\;\times t_{irr}\right)$$

where *k* is the pseudo first order constant of the de-colouring process. *k* can be considered the measure of the photocatalytic SC performance of the tested sample.

The evaluation of the self-cleaning ability of all the photocatalytic materials with MB was carried out using the 660–690 nm integration range. Figure 3B shows the self-cleaning profiles obtained with the MB on the seven tested materials with their exponential fit Equation (4). The comparison of first order constants (*k*) with those obtained using other dyes is reported later. Every photocatalytic material, with the exception of BaSO_4_, showed significant self-cleaning ability. The best activity with MB was observed on TiO_2_ Merck.

The photocatalytic degradation pathway of MB in water solution has been already reported and two different mechanisms have been proposed [43,44,45]. A quick decrease of the maximum intensity and a blue shift of the absorption band were observed. The spectrophotometric evidence and the MS analysis suggested that the MB degradation in solution occurs through both an oxidative attack on the central ring of MB—with consequent loss of the electronic delocalization and quick decolouring [43]—and concurrent N-demethylation of the dimethyl amino groups that gives the gradual spectral shift. The N-demethylation only partially affects the delocalized electronic structure and consequently does not contribute to the rapid decolouring [44,45]. 

In the SC test reported above, and particularly from the reflectance spectra (see for example Figure 3A), it was manifest that at the solid/solid interface, the prevalent photocatalytic MB degradation path was N-demethylation rather than cleavage of the central ring. The principal intermediates, identified through their *m*/*z* ratios and their λ_max_, were azure A (*m*/*z* = 256, λ_max_ = 627 nm), azure C (*m*/*z* = 242, λ_max_ = 615 nm) and thionine (*m*/*z* = 228, λ_max_ = 603 nm). The monodemethylated product, azure B, was not identified because of its rapid transformation into the more stable azure A. The sulfoxide proposed as the main first by-product in solution by Houas et al. [43] was not observed. All of this evidence outlines that the mechanism under solid/solid conditions is different from that under solid/solution conditions, and that the two tests under different conditions are not equivalent. From the observed time evolution of the above by-products as obtained by HPLS-MS (Figure S5), the mechanism of Figure S6 can be hypothesized for solid/solid conditions. The reported mechanisms for N-demethylation already reported for degradation in solution can be accepted [46,47].

### 3.2. Rhodamine B Self-Cleaning Test

Rhodamine B has been often used instead of MB in photocatalytic tests in solution because MB suffers from self-sensitization. This is a confounding parameter that hinders the measurement of the real photocatalytic activity of a material [48]. In addition, rhodamine B has been used in several photocatalytic activity tests [24,25,26], and in standard norms for hydraulic binders [49]. 

Figure S7 shows the reflectance spectra recorded during the RhB self-cleaning test on barite and the corresponding evolution of A_int_ (integration interval 510–550 nm). No evidence of direct photolysis or thermal degradation of RhB on BaSO_4_ surface was observed.

**Figure 4:**
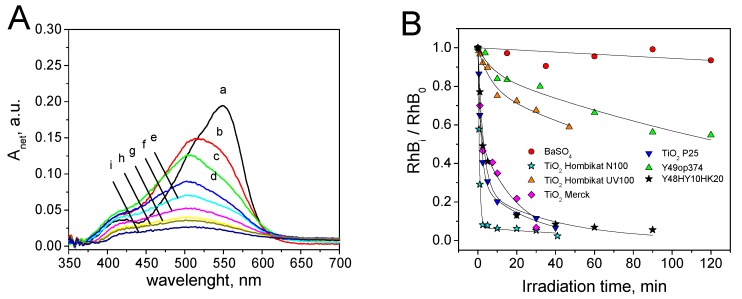
Self-cleaning test with rhodamine B: (**A**) evolution of the surface spectra at different irradiation times (a = 0 min, b = 0.5 min, c = 1 min, d = 2.5 min, e = 5 min, f = 10 min, g = 20 min, g = 30 min, i = 40 min.) during the RhB self-cleaning test on TiO_2_ Merck; (**B**) Time evolution of relative surface concentration observed for RhB on the seven photocatalytic materials; the experimental data were fitted with Equation (5) (double exponential decay) except for BaSO_4_ (single exponential decay). SC = 2 × 10^−5^ g cm^−2^.

Figure 4A shows an example of the spectra recorded during the RhB self-cleaning test on TiO_2_ Merck. With other photocatalytic materials, similar profiles were observed. From the analysis of the spectra, it is manifest that: (*i*) the net absorbance followed a monotonic decrement without significant interferences from coloured by-products; (*ii*) a hypsochromic shift of the peak maximum was observed during the irradiation. 

**Figure 5:**
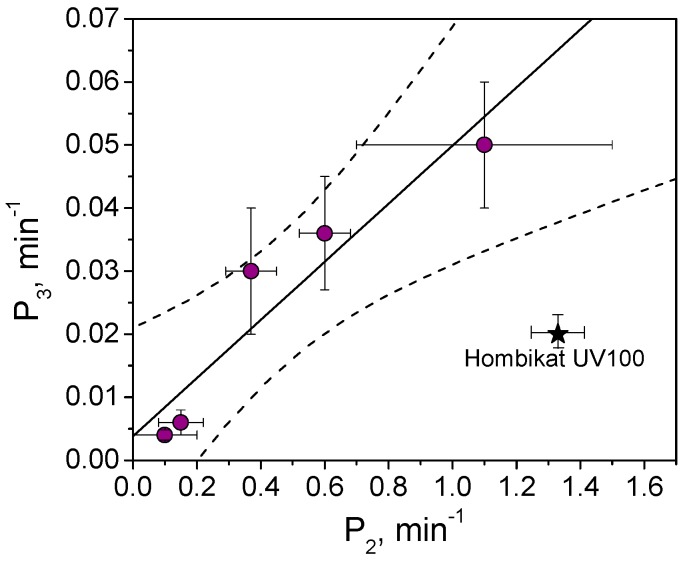
Relationship between the parameters P_3_ and P_2_ obtained from the fit of the rhodamine B relative surface concentration reported in Figure 4B for different photocatalytic materials.

The time evolution of relative surface concentration for the seven tested photocatalytic materials are reported in Figure 4B. With the exception of BaSO_4_ (negligible photocatalytic activity), the colour disappearance was properly described by a double exponential decay (Equation (5)):

(5) $$\frac{RhB_{i}}{RhB_{0}}=\left(1-P_{1}\right)\exp\left(-P_{2}\times t_{irr}\right)+P_{1}\exp\left(-P_{3}\times t_{irr}\right)$$

where *RhB_i_* and *RhB*_0_ are the rhodamine B covering at the time *i* and before irradiation, *P*_1_ is a dimensionless constant in the 0–1 range, *P*_2_ and *P*_3_ are the two first order kinetic constants. Figure S8 shows the fit parameters (*P*_1_, *P*_2_, *P*_3_) obtained for each photocatalytic material. Figure 5 reports the correlation between *P*_2_ and *P*_3_. Please note that: (*i*) the absence of the BaSO_4_ datum, for which a single exponential decay profile with a negligible kinetic constant was observed (see Figure 4B); (*ii*) with the exception of TiO_2_ Hombikat UV100, there is a linear correlation between *P*_2_ and *P*_3_ (r = 0.95, Pearson test passed with α = 0.05) and so both parameters can be used as descriptors of RhB self-cleaning ability. The different self-cleaning profile observed with RhB and MB suggests that RhB was photocatalytic transformed with the kinetic constant *P*_2_ into intermediates, with similar spectra but with lower molar extinction coefficients *ε*, that were then degraded with the second kinetic constant *P*_3_.

### 3.3. Metanil Yellow Self-Cleaning Test

As a consequence of the low optical contrast, the self-cleaning tests with MY were not carried out on the yellow pigments (i.e., with Y49 and Y48HY10HK20, see Table 1). Figure S9 shows the reflectance spectra recorded during the MY self-cleaning test on barite and the corresponding evolution of A_int_ (integration interval 410–440 nm) under irradiation. As for MB and RhB, no evidence of direct photolysis or thermal degradation on BaSO_4_ surface was observed.

**Figure 6:**
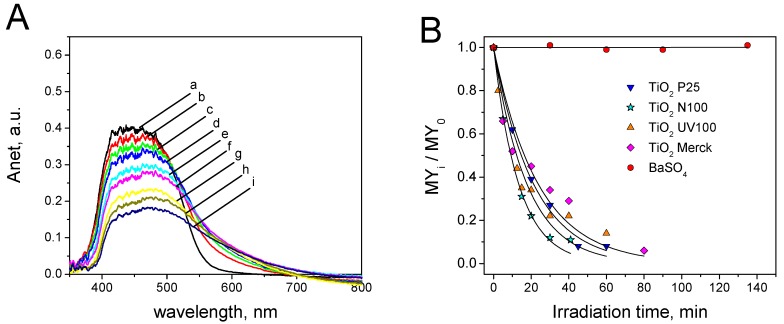
Self-cleaning test with metanil yellow (MY): (**A**) Evolution of the surface spectra as a function of the irradiation time (a = 0 min, b = 10 min, c = 20 min, d = 30 min, e = 45 min, f = 60 min, g = 90 min, g = 120 min, i = 150 min) during the MY self-cleaning test on TiO_2_ Evonik P25; (**B**) Time evolution of relative surface concentration observed for MY on the five tested white photocatalytic materials. SC = 4 × 10^−5^ g·cm^−2^.

Figure 6A shows, as an example, the evolution of the absorbance spectra of MY under irradiation on TiO_2_ Evonik P25. Similar profiles were recorded on the other white samples, with the exception of barite (absence of photocatalytic activity). The surface spectra of MY during the self-cleaning test showed a general monotonic decrease of the spectra intensity in the 400–480 nm and the formation of a shoulder at higher wavelength. This can be attributed to the formation of a coloured intermediate with a red-shifted maximum of absorption. Considering the contribution to the absorption at 411 nm was completely due to MY, it was possible to separate the spectral contribution of MY from that of the main by-product. Figure S10A shows the deconvolution of the spectra reported in Figure 6A. The spectrum of the main intermediate shows a maximum at 544 nm. Figure S10B reports the evolution of the net absorbance at 544 nm for the intermediate as a function of the irradiation time. A bell shaped profile was observed as a consequence of the formation of the intermediate and its subsequent photocatalytic degradation. Figure S10C shows the decolouring profiles considering or not the contribution of the intermediate (SC test on TiO_2_ P25). The difference between the two profiles was not significant. So, the photocatalytic formation of the main intermediates observed did not affect the decolouring profile and subsequently the evaluation of the self-cleaning properties of the tested samples.

Figure 6B shows the time-evolution of relative surface concentrations. The self-cleaning profiles followed a single exponential decay (see Equations (4)). The kinetic constants *k* are then a direct measure of the self-cleaning performance of the tested photocatalytic material. They are reported later. For the tested photocatalytic materials, the following order of SC activity was observed: TiO_2_ Hombikat N100 > TiO_2_ Hombikat UV100 > TiO_2_ Evonik P25 > TiO_2_ Merck >> barite.

The degradation of MY was also studied in aqueous suspension to ascertain if there are differences between the photocatalytic degradation under solid/solid and solid/electrolyte conditions. The degradation of MY in solution in the presence of the white photocatalytic materials was carried out and the decrease of absorbance was followed spectrophotometrically (λ = 411 nm). 

Figure S11A shows, as an example, the spectra of the filtered solutions at different irradiation times in the presence of TiO_2_ Hombikat N100. Similar results were observed in the presence of the other photocatalytic materials with the exception of BaSO_4_. On barite, no change of the spectra was recorded with the irradiation time due to the absence of photocatalytic activity, and of thermal and direct photochemical degradation of the dye. The spectra of the filtered suspension at different irradiation times (see Figure S11A) showed a monotonic decrease of the overall intensity without accumulation of other coloured by-products in the aqueous phase. During the MY photocatalytic degradation in solution with TiO_2_ Evonik P25, the semiconductor was collected on 0.45 μm filter after different irradiation times and the reflectance spectrum of each filter was also recorded. The evolution of the net absorbance *A_net_* at different irradiation times is reported in Figure S12 (the spectrum of the pristine filter was used as background). The evolution of *A_net_* showed the formation of by-products adsorbed at the surface, which gave a brownish appearance to the photocatalyst. It is worth noting that the ortho-dihydroxilated aromatic rings give stable brown complexes with Ti(IV) ion at the surface [50]. Conversely, the formation of brownish compounds was not observed at the solid/solid interface. A different mechanism of degradation in water suspension and at the solid/solid interface could be envisaged.

**Figure 7:**
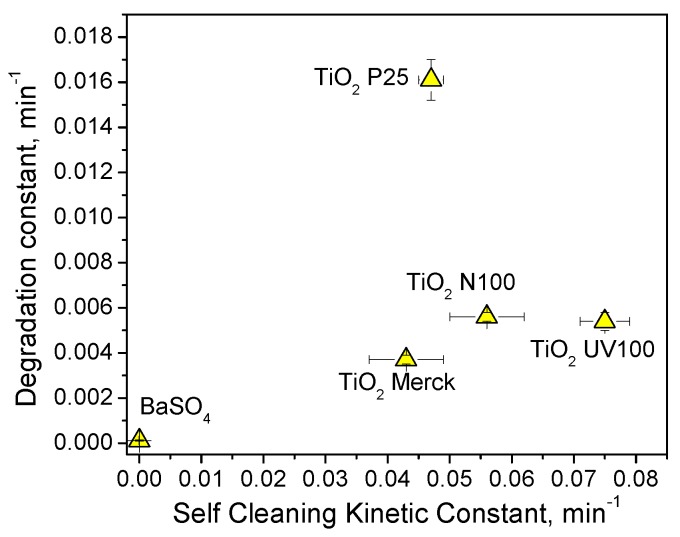
Comparison between the MY degradation kinetic constant measured in aqueous solution and the photocatalytic self-cleaning kinetic constants in the presence of the five white photocatalytic materials.

Figure S11B shows the disappearance of MY in solution with the tested photocatalytic materials. The degradation of MY in the experimental conditions followed a pseudo-first order kinetic. The related kinetic constants are reported in Figure S11C. The order of photoactivity in aqueous solution was: TiO_2_ Evonik P25 > TiO_2_ Hombikat UV100 > TiO_2_ Hombikat N100 > TiO_2_ Merck >> barite. This order was significantly different from that of the self-cleaning activity (see above). Figure 7 shows the comparison between the degradation kinetic constants measured in aqueous solution and the photocatalytic self-cleaning kinetic constants evaluated on the tested white photocatalytic materials. There was no direct correlation between the activity in solution and that at the solid/solid interface. This experiment highlighted that the evaluation of the photocatalytic activity should always be carried out in the real operative conditions, i.e., for the self-cleaning properties at the solid/solid interface and not in aqueous suspensions.

### 3.4. Comparison among the Results Obtained by Using the Three Dyes

**Figure 8:**
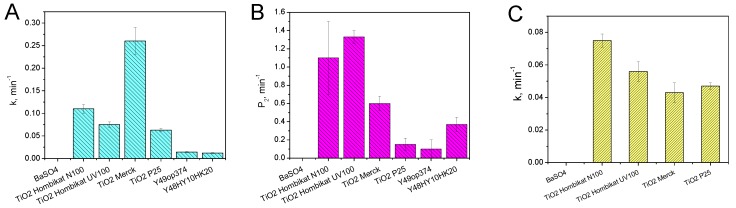
Summary of the photocatalytic self-cleaning kinetic constants measured on the tested materials with methylene blue (**A**), rhodamine B (P_2_ parameter) (**B**) and metanil yellow (**C**).

Figure 8 reports the summary of observed kinetic constants in the SC tests with the 3 dyes. It is clearly evident that the photocatalytic materials have different reactivity with the different dyes. As an example, TiO_2_ Merck showed high reactivity toward MB, while lower self-cleaning activity with MY and RhB. This selectivity is not surprising, as it has already been observed with slurries. Substrate selectivity in solution has been previously reported by Ryu and Choi [51], who compared the photoactivity in water of different commercial titanium dioxides toward 19 organic and inorganic substrates. They observed a marked selectivity of some photocatalysts toward specific families of substrates emphasizing that the photocatalytic activity is strongly related to the substrate nature. This selectivity is a drawback for SC tests, as it reflects in a discrimination depending on the chosen dye. As TiO_2_ Merck seems to be highly selective toward MB, a SC test that used MB only would overestimate the SC properties of the cited catalyst. Conversely, TiO_2_ Hombikat N100 would result in a very good catalyst when tested with RhB and MY, whilst it would have poor SC properties if tested with MB. Then the use of three dyes in the self-cleaning test allows not only the selection of best optical contrast with the substrate, but also avoids a methodological bias that leads to over- or under-estimation in the case of peculiar substrate selectivity.

A principal component analysis (PCA) of data reported in Figure 8 is reported in Figure S13. The PC1 reflects mainly the SC activity. Then, TiO_2_ Hombikat UV100 and N100, together with TiO_2_ Merck, are the most active materials. These materials are separated on PC2, which reflects the selectivity toward a given dye. In addition, the loadings of MB and RhB are almost orthogonal, indicating the absence of correlation between the data obtained with these dyes. MY is quite correlated with RhB, with low correlation with MB. This outlines that a test with a single dye can be biased by a selective reactivity with a specific photocatalytic material. Consequently, considering the optical contrast, an informative test would imply the use of at least two dyes. 

## 4. Conclusions

The SC test applied here was simple and applicable without the use of complex analytical tools. It requires the measurement of the reflectance spectra of the tested surface. Following the reflectance spectrum during the decolourization, it was possible to select the proper spectral window where the interference from the by-product is minimized. Under these conditions the measured integrated absorbance follows an exponential decay for MB and MY. Instead, RhB showed a double exponential decay, where the two decay constants were well correlated. The conditions simulated the real environment for self-cleaning work for photocatalytic materials (solid/solid interface).

The time evolution of reflectance spectra gave insights into the photocatalytic transformation of the dyes at the solid/solid interface. The study of the by-products of MB showed that, different to slurries where the degradation occurs mainly with the cleavage of the central ring, at the solid/solid interface, the N-demethylation of the two symmetric dimethyl amino groups prevails. Also the MY degradation at the solid/solid and solid/electrolyte interface showed a different order of reactivity. Then, for evaluating the self-cleaning performance, the experimental data underlined the importance of carrying out experiments at the solid/solid interface rather than tests in solution. The correlations between the self-cleaning activity and the physico-chemical properties of the powders, as well as the chemical and photochemical mechanisms operational during the self-cleaning process on very different materials, were not further explored because the aim of the work was mainly to test the significance of the procedure. Some cases of substrate selectivity of the tested photocatalytic materials toward the organic dyes were observed. This encourages the use of more than one dye for evaluating the self-cleaning ability of a photocatalytic material with the aim to avoid discrimination among the samples.

## Figures and Tables

**Table 1:** Test Samples. (*l* = length, *w* = width).

Sample Name/Producer	Material	Average Particle Size, nm
BaSO_4_ (Rockwood Italia S.p.A.)	Barite	700–1000
TiO_2_ Hombikat N100 (Sachtleben Chemie GmbH)	Titania (Anatase)	20
TiO_2_ Hombikat UV100 (Sachtleben Chemie GmbH)	Titania (Anatase)	10
TiO_2_ Merck	Titania (Anatase)	40–300
TiO_2_ P25 (Evonik)	Titania (Anatase 80%, Rutile 20%)	40
Y49 (Rockwood Italia S.p.a.)	Goethite	Acicular shape *l* = 1000–1300 *w* = 100
Y48HY10HK20 (Rockwood Italia S.p.a.)	Goethite	Acicular shape *l* = 1500–2000 *w* = 100–200

**Table 2:** Dyes for the photocatalytic self-cleaning test: standard dye covering (SC) calculated on the geometric sprayed area of the sample, wavelength of maximum absorbance, molar extinction coefficient in acetone and interval of wavelength over which absorbance was integrated to calculate the integrated absorbance A_int_ and the calibration function for the dye covering (DC).

Dye	Standard Covering (SC)		Molar Extinction Coefficient at λ_max_ (M^−1^·cm^−1^)	Maximum Absorbance (λ_max_, nm)	Interval of Absorbance Integration (nm)
	(g·cm^−2^)	(molecule·cm^−2^)			
Methylene Blue (MB)	4 × 10^−5^	6.4 × 10^16^	23,430	657	660–690
Rhodamine B (RhB)	2 × 10^−5^	2.5 × 10^16^	60,750	556	510–550
Metanil Yellow (MY)	4 × 10^−5^	3.2 × 10^16^	21,750	411	410–440

## Supplementary Materials

The following are available online at http://www.mdpi.com/1996-1944/12/3/508/s1, Figure S1: Absorption spectra in the 350–750 nm range of 2 × 10^−5^ M solution of Methylene Blue, Rhodamine B and Metanil Yellow in Acetone (cuvette path length 10 mm). Figure S2: Absorbance spectra of MB (A), RB (B) and MY (C) over the different tested material before irradiation. Standard Covering 4 × 10^−5^ g·cm^−2^ for MB and MY and 2 × 10^−5^ g·cm^−2^ per RB. Figure S3: Emission spectrum in the 200–800 nm range of the fluorescence lamps used during the self-cleaning tests (Philips TLK 40W/05). Figure S4: Methylene blue self-cleaning test on BaSO_4_: reflectance spectra (net absorbance, A_net_) in the 300–800 nm range at different irradiation time. SC = 4 × 10^−5^ g·cm^−2^. Figure S5: Methylene blue self-cleaning test on Hombikat N100: time evolution of the peak area for the three main intermediates. The peak areas were obtained extracting from the Total Ion Current (TIC) the signal of the single ions with *m*/*z* 228, 242 and 256. SC = 4 × 10^−5^ g·cm^−2^. Figure S6: First steps of the MB photocatalytic degradation pathway at the solid/solid interface. Figure S7: Rhodamine B self-cleaning test on BaSO_4_: A) reflectance spectra (net absorbance, A_net_) in the 300–800 nm range at different irradiation time; B) decolouring profile (DC computed in the 510–550 nm range). Initial dye covering 2 × 10^−5^ g·cm^−2^. SC = 2 × 10^−5^ g·cm^−2^. Figure S8: Rhodamine B self-cleaning test on different white and yellow samples: fit parameters (*P*_1_, *P*_2_, *P*_3_) obtained from the decolouring profiles for each self-cleaning experiment. Figure S9: Metanil Yellow self-cleaning test on BaSO_4_: A) reflectance spectra (net absorbance, A_net_) in the 350–800 nm range at different irradiation time; B) decolouring profile (DC computed in the 410–440 nm range). SC = 4 × 10^−5^ g·cm^−2^. Figure S10: Self-cleaning test with Metanil Yellow on TiO_2_ P25: A) deconvolution of the surface spectra for the contribution due to the principal coloured by-product; B) absorbance at 554 nm for the component related to the by-product as a function of the irradiation time; C) decolouring self-cleaning profiles obtained considering (*k’*) and not (*k*) the contribution of the coloured by-product. SC = 4 × 10^−5^ g·cm^−2^. Figure S11: Photocatalytic Degradation of Metanil Yellow in aqueous solution: A) spectra of the filtered solutions at different irradiation time in the presence of TiO_2_ Hombikat N100 (irradiation times: a = 0 min; b = 1 min; c = 3 min; d = 6 min; f = 10 min; g = 20 min; h = 30 min; i = 40 min; j = 60 min; k = 80 min; a = 120 min; l = 160 min); B) degradation profiles observed with the five tested white powders; C) summary of the photocatalytic degradation kinetic constants. Figure S12: Evolution of the reflectance spectra in A_net_ of the photocatalyst collected on 0.45 µm filters during the degradation test of MY with TiO_2_ P25 in aqueous solution (irradiation times: a = 0 min; b = 1 min; c = 3 min; d = 6 min; e = 10 min; f = 20 min; g = 40 min; h = 80 min; i = 120 min). Figure S13: Biplot graph of scores and loadings resulting from the Principal Component Analysis (PCA) of the data reported in Figure 8—carried out with the free chemometric software V-Parvus 2008. The data were autoscaled and normalized over their variance. The cumulative variance on the first two Principal Components was 93.3%.
