# Supplementary material for: Quantification of the Photocatalytic Self-Cleaning Ability of Non-Transparent Materials
Source: Materials (Basel). 2019 Feb 8;12(3):508. doi: 10.3390/ma12030508 (PMC6384723; doi:10.3390/ma12030508)
Supplement: Supplementary file 1 [file materials-12-00508-s001.pdf]

materials

Supplementary Information

Article

Quantification of the Photocatalytic Self-Cleaning Ability of Non-Transparent Materials

Marco Minella and Claudio Minero *

Department of Chemistry and NIS Center of Excellence, University of Torino, Via P. Giuria 5, Torino 10125, Italy; marco.minella@unito.it

* Correspondence: claudio.minero@unito.it; Tel.: +39-011-6708449

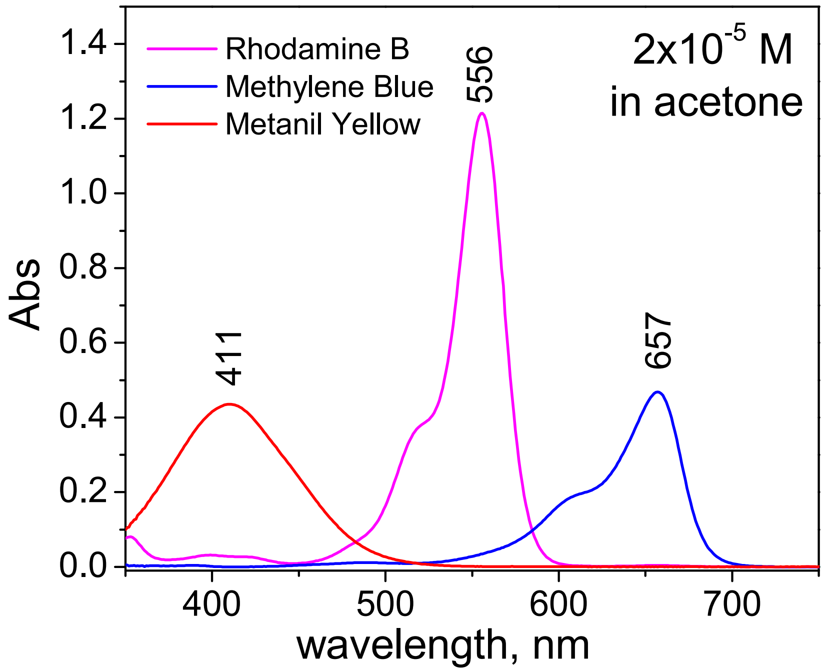

Figure S1. Absorption spectra in the 350-750 nm range of 2×10^{-5} M solution of Methylene Blue, Rhodamine B and Metanil Yellow in Acetone (cuvette path length 10 mm).

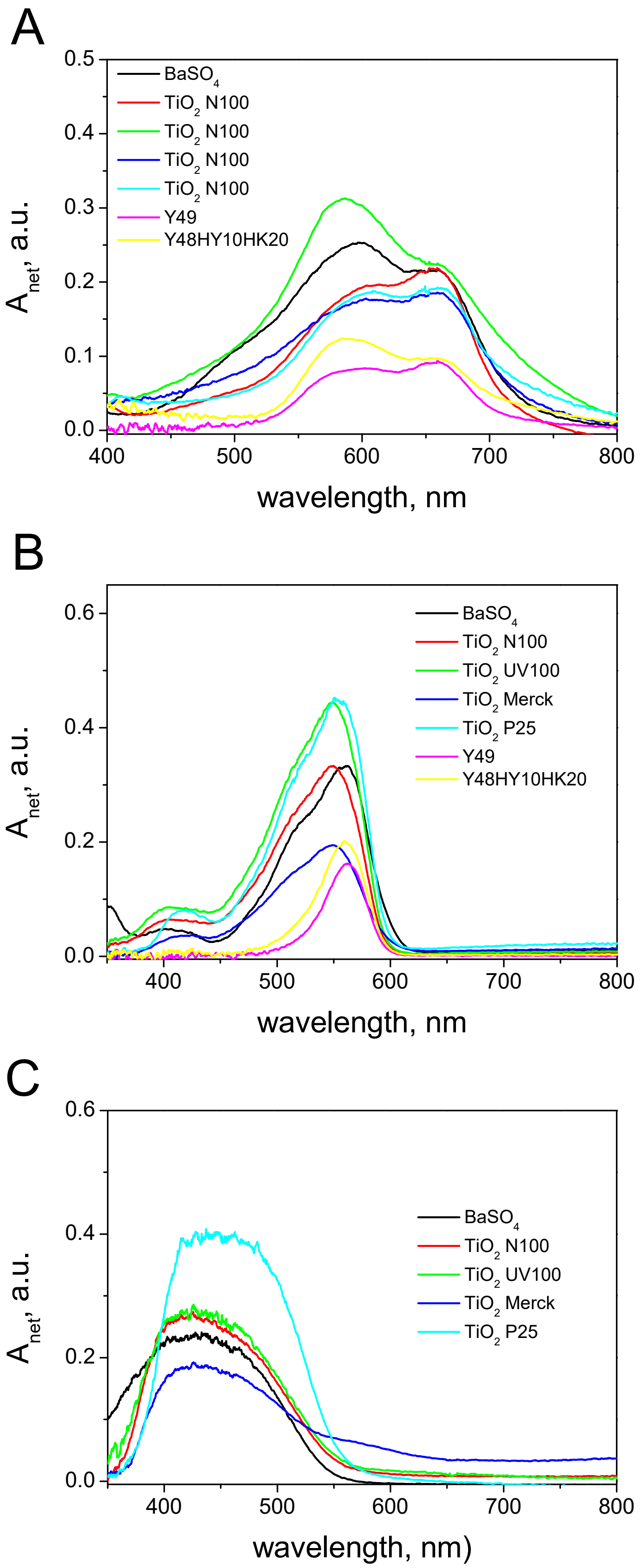

Figure S2. Absorbance spectra of MB (A), RB (B) and MY (C) over the different tested material before irradiation. Standard Covering 4×10^{-5} g·cm^{-2} for MB and MY and 2×10^{-5} g·cm^{-2} per RB.

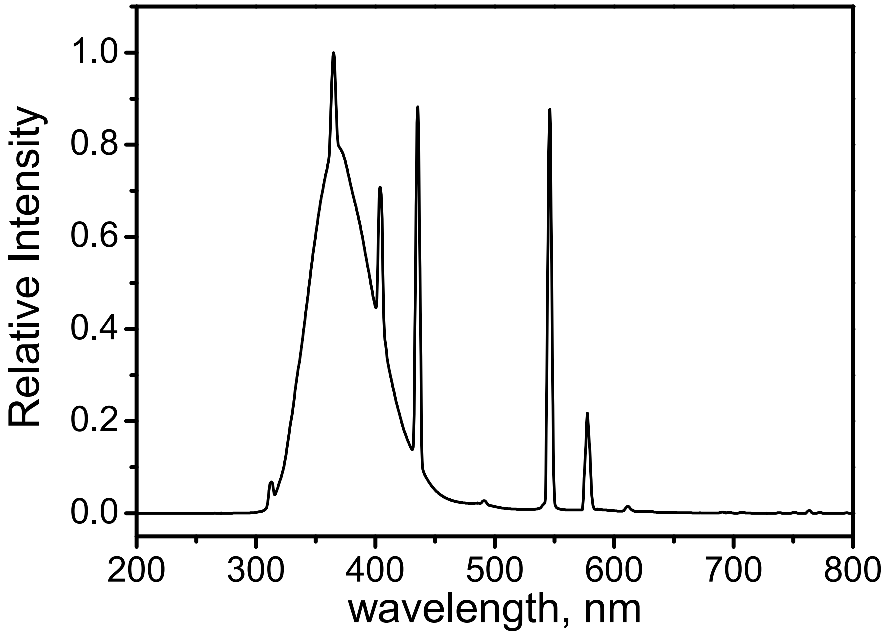

Figure S3. Emission spectrum in the 200-800 nm range of the fluorescence lamps used during the self-cleaning tests (Philips TLK 40W/05).

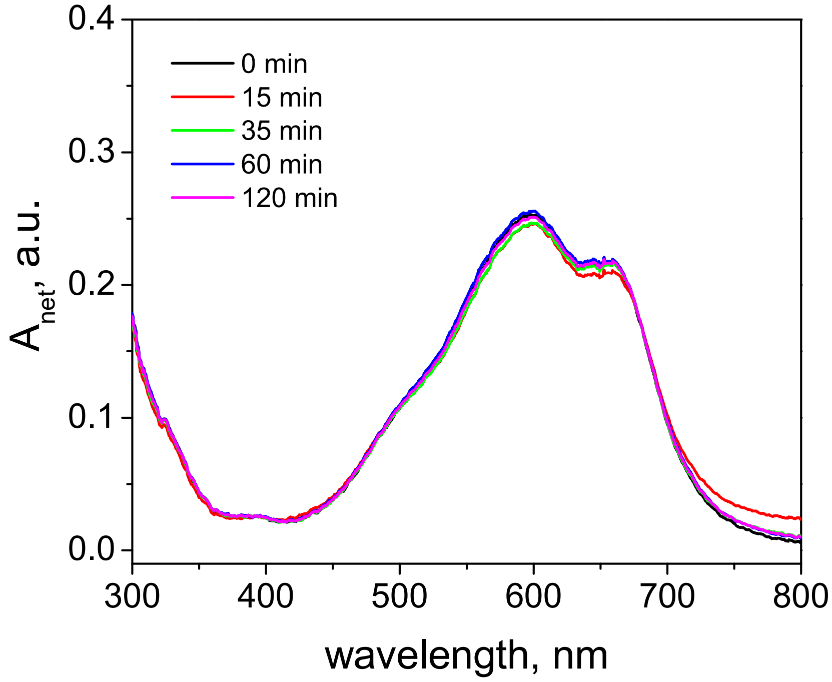

Figure S4. Methylene blue self-cleaning test on $BaSO_4$: reflectance spectra (net absorbance, A_{net}) in the 300-800 nm range at different irradiation time. SC = 4×10^{-5} g·cm^{-2}.

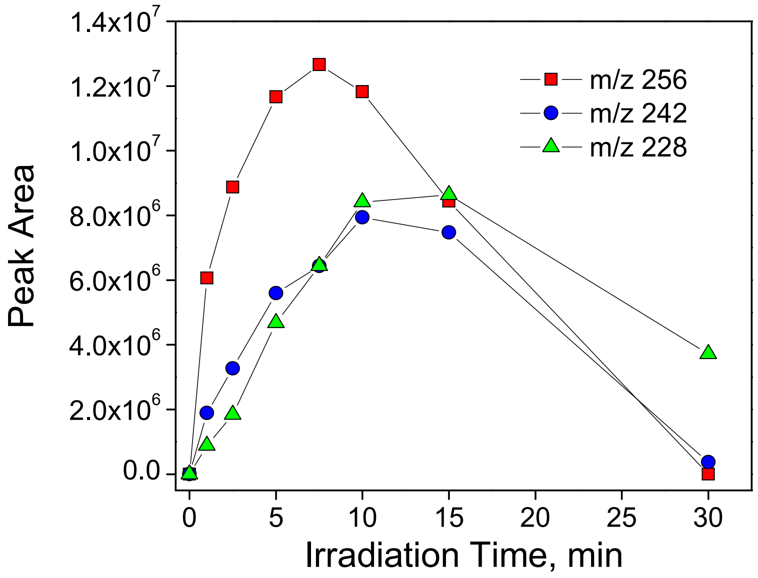

Figure S5. Methylene blue self-cleaning test on Hombikat N100: time evolution of the peak area for the three main intermediates. The peak areas were obtained extracting from the Total Ion Current (TIC) the signal of the single ions with m/z 228, 242 and 256. SC = 4×10^{-5} g·cm^{-2}.

A)

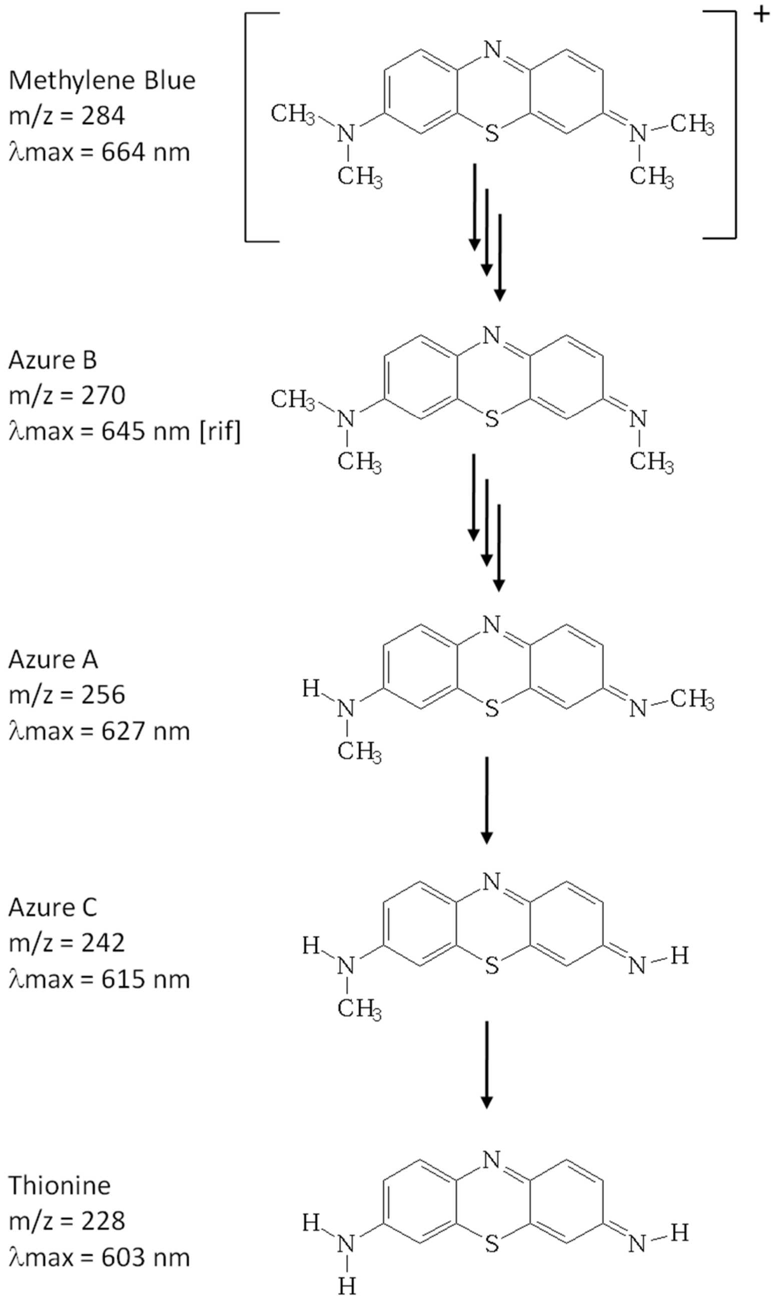

Figure S6. First steps of the MB photocatalytic degradation pathway at the solid/solid interface

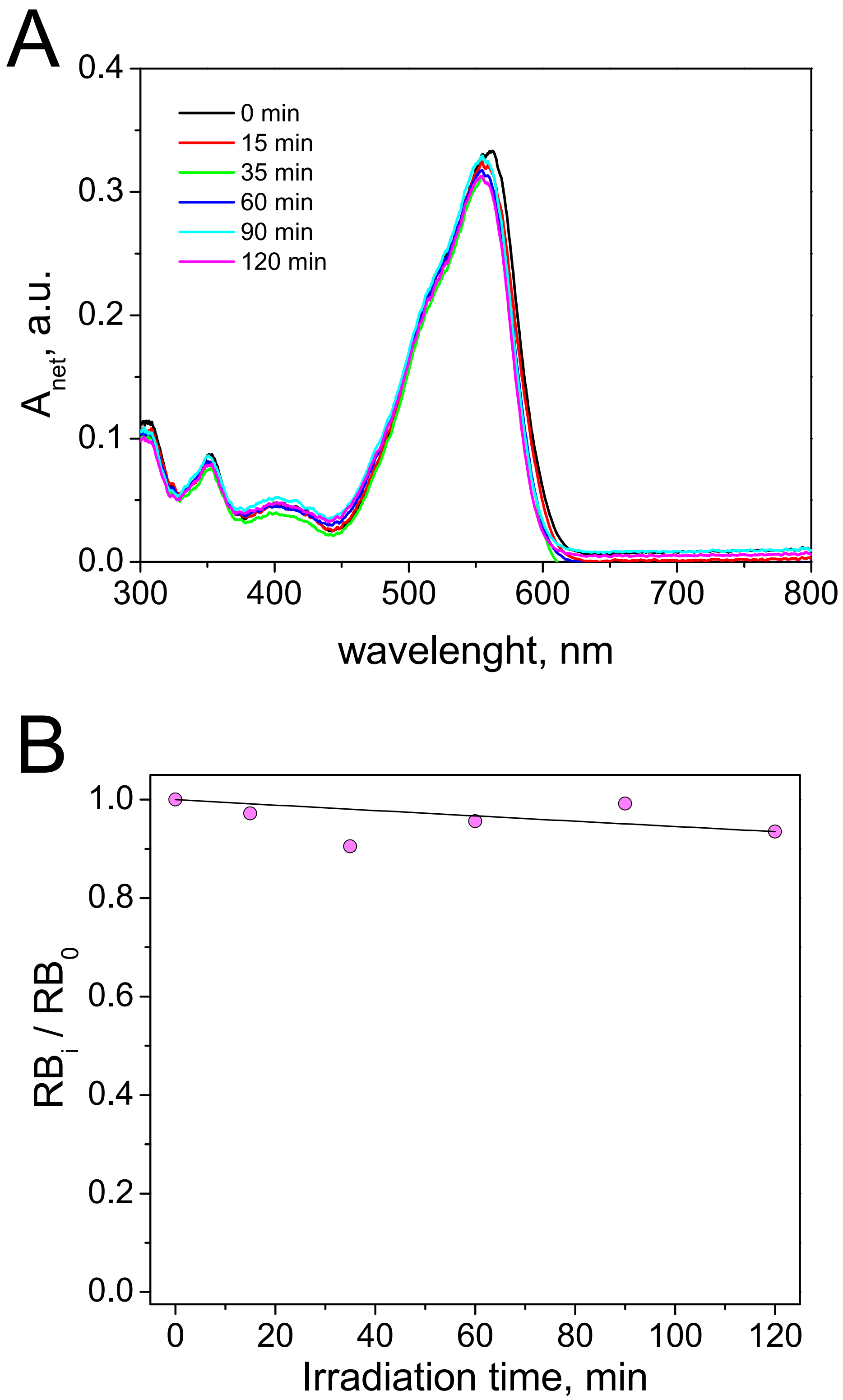

Figure S7. Rhodamine B self-cleaning test on $BaSO_4$: A) reflectance spectra (net absorbance, A_{net}) in the 300-800 nm range at different irradiation time; B) decolouring profile (DC computed in the 510-550 nm range). Initial dye covering $2{\times}10^{-5}$ g·cm^{-2}. SC = $2{\times}10^{-5}$ g·cm^{-2}.

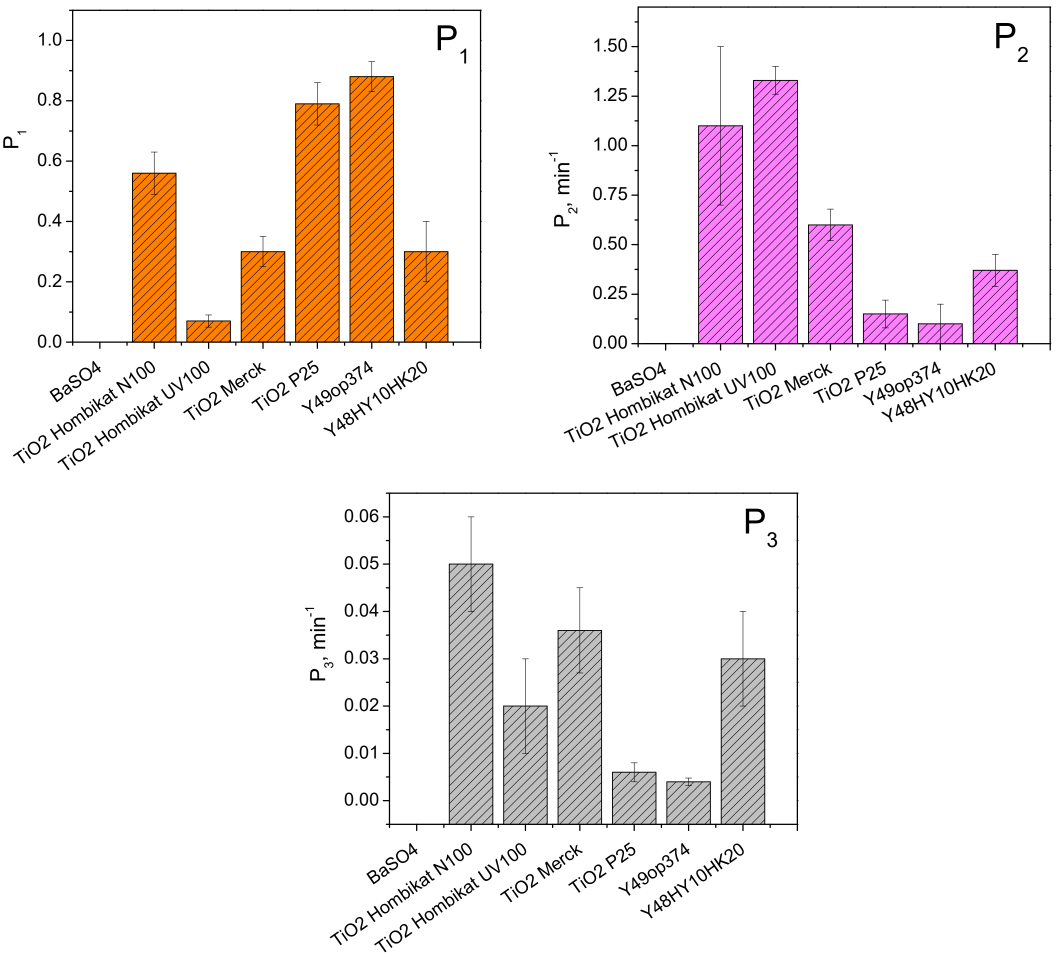

Figure S8. Rhodamine B self-cleaning test on different white and yellow samples: fit parameters (*P_1*, *P_2*, *P_3*) obtained from the decolouring profiles for each self-cleaning experiment.

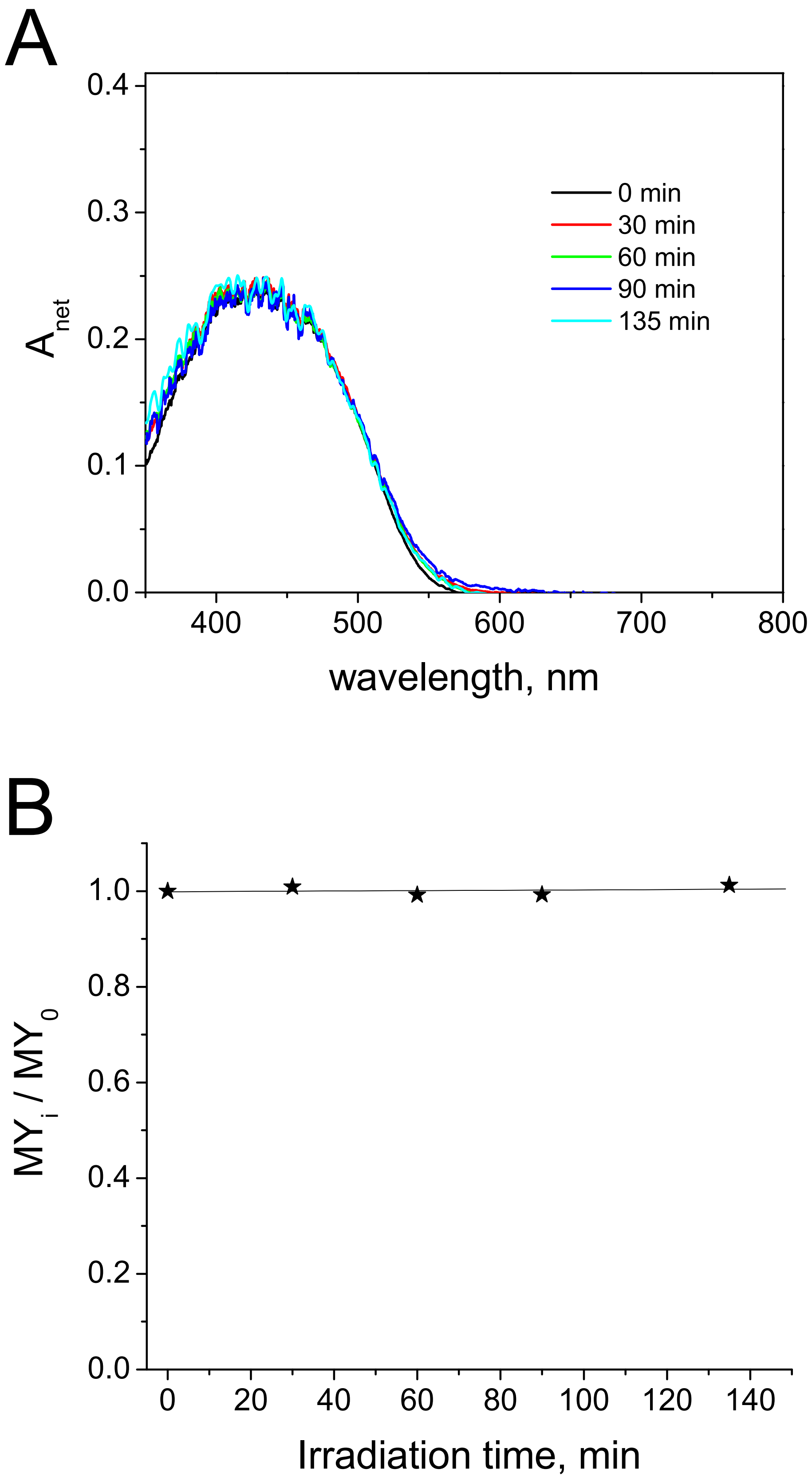

Figure S9. Metanil Yellow self-cleaning test on $BaSO_4$: A) reflectance spectra (net absorbance, A_{net}) in the 350-800 nm range at different irradiation time; B) decolouring profile (DC computed in the 410-440 nm range). SC = 4×10^{-5} g·cm^{-2}.

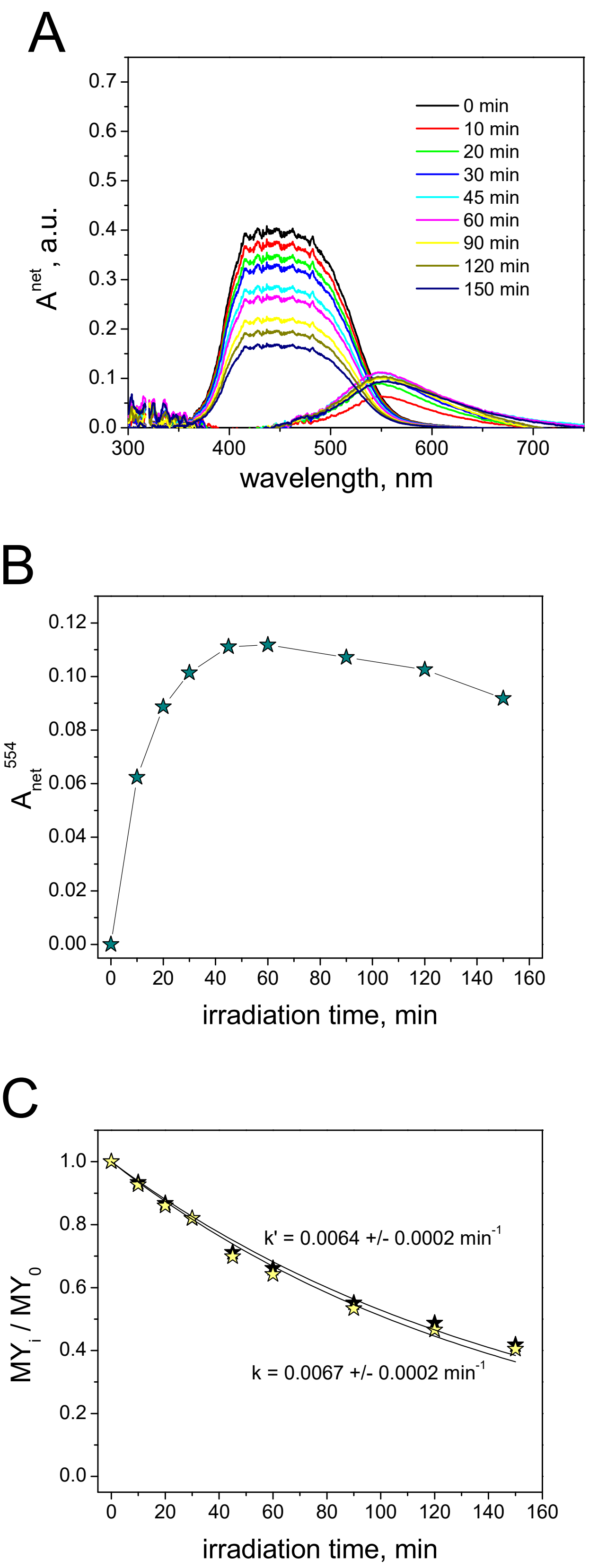

Figure S10. Self-cleaning test with Metanil Yellow on TiO_2 P25: A) deconvolution of the surface spectra for the contribution due to the principal coloured by-product; B) absorbance at 554 nm for the component related to the by-product as a function of the irradiation time; C) decolouring self-cleaning profiles obtained considering (*k′*) and not (*k*) the contribution of the coloured by-product. SC = 4×10^{-5} g·cm^{-2}.

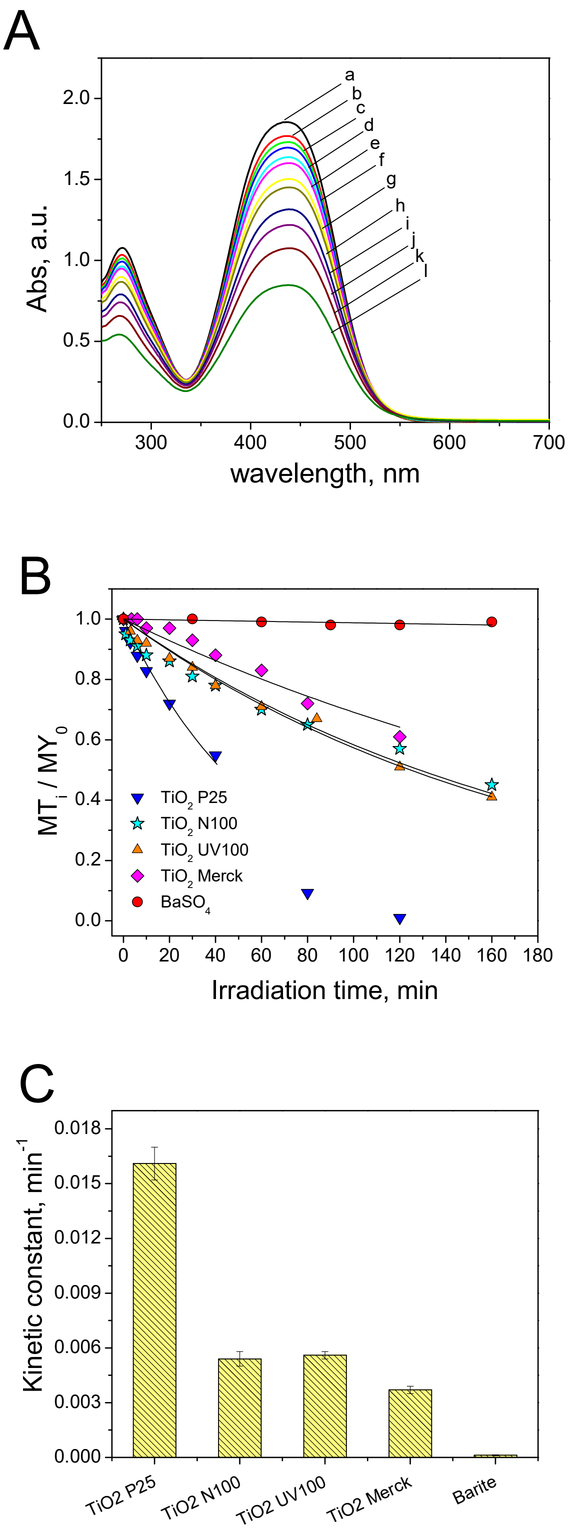

Figure S11. Photocatalytic Degradation of Metanil Yellow in aqueous solution: A) spectra of the filtered solutions at different irradiation time in the presence of TiO_2 Hombikat N100 (irradiation times: a=0 min; b=1 min; c=3 min; d=6 min; f=10 min; g=20 min; h=30 min; i=40 min; j=60 min; k=80 min; a=120 min; l=160 min); B) degradation profiles observed with the five tested white powders; C) summary of the photocatalytic degradation kinetic constants.

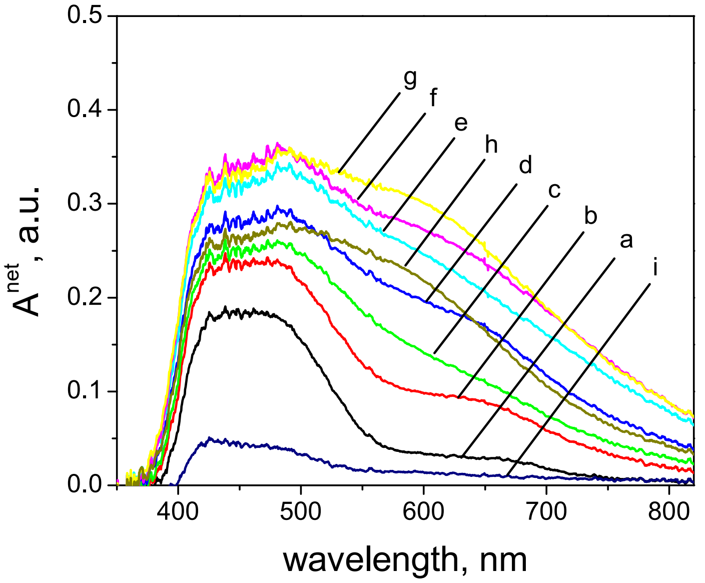

Figure S12. Evolution of the reflectance spectra in A_{net} of the photocatalyst collected on 0.45 μm filters during the degradation test of MY with TiO_2 P25 in aqueous solution (irradiation times: a=0 min; b=1 min; c=3 min; d=6 min; e=10 min; f=20 min; g=40 min; h=80 min; i=120 min).

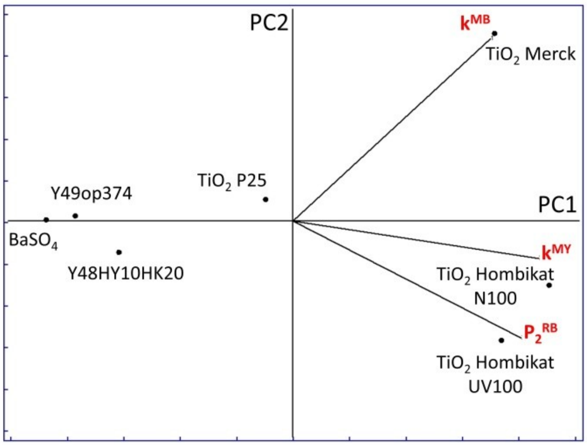

Figure S13. Biplot graph of scores and loadings resulting from the Principal Component Analysis (PCA) of the data reported in Figure 8. – carried out with the free chemometric software V-Parvus 2008 [i]. The data were autoscaled and normalized over their variance. The cumulative variance on the first two Principal Components was 93.3%.

---

i . Forina, M.; Lanteri, S.; Armanino, C.; Casolino, C.; Casale, M.; Olivieri, P. V-Parvus. 2008. http://www.parvus.unige.it.
